# Liquid chromatography fingerprint analysis and antioxidant activity of selected lavender species with chemometric calculations

**DOI:** 10.1371/journal.pone.0218974

**Published:** 2019-07-09

**Authors:** Anna Hawrył, Mirosław Hawrył, Monika Waksmundzka-Hajnos

**Affiliations:** Department of Inorganic Chemistry, Faculty of Pharmacy, Medical University of Lublin, Lublin, Poland; College of Agricultural Sciences, UNITED STATES

## Abstract

The extracts of seven *Lavandulae* species (*Lavandula stoechas*, *Lavandula lanata*, *Lavandula viridis*, *Lavandula angustifolia „Rosea”*, *Lavandula angustifolia „Afropurpurea”*, *Lavandula angustifolia* and one unknown) were analyzed using the reversed-phase-high performance liquid chromatography–diode array detection (RP-HPLC–DAD) with gradient elution technique to obtain the chromatographic fingerprint profiles. The HPLC analysis was performed using the Kinetex RP18 chromatographic column and eluent consisting of methanol-water-0.1% formic acid (5–100% (v/v)) at 30 °C with the run time of 60 min. and the detection wavelength 280 nm. The chromatograms were preliminary processed with the smoothing, noise reduction, background subtraction and alignment using the SpecAlign program (version 2.4.1). The presence of selected standards (apigenin, myricetin, luteolin, luteolin 7-glucoside, chlorogenic acid, caffeic acid, ferulic acid) in the extracts was confirmed. The chemical similarity between studied plants was evaluated using the Cluster Analysis (Pearson correlation coefficient, r, and Euclidean) and PCA. The preliminary antioxidant activity of studied extracts was evaluated based on the total phenolic content (Folin-Ciocalteu method), ferric ion reducing antioxidant parameter (FRAP) and α,α-diphenyl-β-picrylhydrazyl (DPPH) free radical scavenging method using the spectrophotometric technique.

## Introduction

*Lavandulae* species belongs to *Lamiaceae* family and includes about 39 species and they are cultivated mainly in Europe. The genus *Lavandula* are traditionally used for the treatment of depression, headache, stress, migraine and diabetes [1,2]. Due to presence of the essential oils (monoterpenes) lavender has an antimicrobial, antifungal, antiflatulence, antiholic and carminative effects and is used in the cosmetics industry [1,3–5]. Moreover, the *Lavandulae* species are also rich in the secondary metabolites, such as phenolic acids, flavonoids, coumarins, diterpenes, triterpenes, tannins [2,6,7]. Due to the presence of the phenolic compounds the lavender extracts show the antioxidant activity [8–11].

One aspect of the chemical quality evaluation of a herbal medicine is the development of chemical fingerprint, which has been introduced and accepted by WHO, as a strategy for quality estimation of medicinal plants [11–13]. There are a lot of analytical methods (e.g. GC, HPLC, LC, HPTLC), but the most popular and rapid fingerprint technique is HPLC [14–18].

The chemical composition of the lavender extracts or essential oils were studied using the HPLC analysis. The phenolic composition of the infusion or decoction of sea lavender (*Lavandulae algarvense*) was analyzed to confirm the antioxidant and antidiabetic activities [19]. The impact of different storage conditions on the quality of lavender essential oils was also studied using, among others, this method [20]. In another work the phenolic profiles screening aromatic *Lamiaceae* species (among others *Lavandula officinalis*) were constructed using a novel and validated ultra performance liquid chromatography (UPLC) method coupled with DAD diode array detector and tandem mass spectrometry (MS/MS) in negative mode of electrospray ionization [21]. Phytochemical analysis of hydromethanolic *Lavandulae* extracts (*L*. *stoechas* and *L*. *dentate*) was performed using Reversed Phase-High Performance Liquid Chromatography-Diode Array Detector- Quadrupole-time-offlight-Mass Spectrometry (RP–HPLC–DAD–QTOF–MS) for the evaluation of their qualitative and quantitative differences [22]. The HPLC-DAD analysis of selected lavenders ((*Lavandula officinalis* Chaix, *L*. *spica* Lois, *L*. *vera* D. C., *L*. *angustifolia* Mill. ssp. *angustifolia*, or *L*. *officinalis* Chaicah) was also performed to quality control of the species for detection of some phenolic compounds [6]. Similarly, some phenolics (phenolic acids) of *Lavandula angustifolia* Mill. (LA) extract were analyzed by LC-MS to confirm its biological activity [23]. The essential oil of *Lavandula angustifolia* MILL. was also examined using the HPLC [20] or HPLC-DAD-MS [24,25] methods.

The chromatographic fingerprint herbal profile can be defined as characteristic signals for selected plants and the obtained chromatograms can be compared to unambiguously identify the sample [26]. The HPLC fingerprint analysis has been accepted by WHO as method for the chemical evaluation of herbal medicines [27]. The data of HPLC fingerprint chromatograms are extensive and therefore some chemometric techniques can be used to the evaluation of the chemical similarities and differences between studied samples [15,16].

The aim of this study is RP-HPLC–DAD gradient elution analysis with the chemometric calculations of selected lavenders with confirmation of their identity and one unknown plant. Moreover, the antioxidant activity of *Lavandulae* extracts was calculated based on the total phenolic content using the Folin-Ciocalteu [28], FRAP [29] and DPPH [30] methods; all techniques were performer using the spectrophotometric methods.

The fingerprint chromatographic data combined with the antioxidant activity can allow to create a multivariate regression model, which can be used to provide a scientific way for the quality control of plant materials [31–33].

## Materials and methods

### Extraction procedure

Six known and one unknown *Lavandulae* species (Table 1) were collected in Botanical Garden of Maria Curie-Skłodowska University (Lublin, Poland) and the identity of plants was confirmed by worker of them. The voucher specimen was also from this Botanical Garden. Dried (in the air) plant materials were pulverized in the hand mill. Next, the obtained samples were weighted (about 5 g, according to Table 1), located in the paper cases and placed in 250 mL flat bottom flask. Next, the 150 mL of the xylene (Merck, Darmstadt, Germany) was added and the flasks were located in the ultrasonic bath (Bandelin RK 100 H,Heidolph, Schwabach, Germany) under reflux for 4 hours. The obtained xylene extracts were evaporated under reduced pressure using the vacuum evaporator (Heidolph, Schwabach; Laboxact, KNF, Freiburg im Breisgau; Germany). The dried (at room temperature) xylene extracts were dissolved in 25 mL graduated flasks in the xylene. Next, the samples (dried paper cases) were placed in the 250 mL flat bottom flask, and the 250 mL of methanol was added and located in ultrasonic bath under reflux for the 4 hours. The methanolic extracts were evaporated under reduced pressure using the vacuum evaporator. After that, the dried methanolic extracts were dissolved in methanol in 25 mL graduated flasks. Xylene and methanolic extracts were stored in a refrigerator (4°C).

**Table 1:** The names of *Lavandula* species and the weight of raw material.

Number of extract	*Lavandulae* species	Weight [g]
1	*Lavandula stoechas*	5.0160
2	*Lavandula lanata*	5.0080
3	*Lavandula viridis*	5.0206
4	*Lavandula angustifolia „Rosea”*	5.0046
5	X	5.0022
6	*Lavandula angustifolia „Afropurpurea”*	5.0089
7	*Lavandula angustifolia*	5.0039

### High Performance Liquid Chromatography (HPLC) apparatus and reagents

The HPLC gradient elution analysis was performed on Merck-Hitachi LaChrom Elite System (Merck, Darmstadt, Germany) with diode array detector L-2455, thermostat L-2300, pump L-2130 and autosampler L-2200 with the Kinetex RP18 (5 μm, 150 × 4.6 mm) chromatographic column (Phenomenex, Torrance, CA, USA) as stationary phase. The mobile phase was consisted of methanol-water-0.1% formic acid (gradient 5–100% (v/v) of methanol) at 30 °C and the run time was 60 min. The analysis was performed with the detection wavelength 280 nm, the sample injection volume was 10 μL and the flow rate was set to 1.0 mL min^−1^. Methanol Chromasolv for HPLC was purchased from Sigma-Aldrich (St. Louis, MO, USA) and formic acid from Polish Reagents (Polish Reagents, Gliwice, Poland); double-distilled water was used.

### Preparation of standards

Samples of standards (Sigma-Aldrich, Saint Louis, USA) were weighted and dissolved in methanol (Polish Reagents, Gliwice, Poland) to obtain 0.1% solutions. The retention time values obtained for the selected standards in used chromatographic gradient elution system were presented in Table 2.

**Table 2:** The name of standards and their retention time values.

Name	Abbreviation	Retention time
apigenin	Ap	35.167
myricectin	Myr	27.013
luteolin	Lt	32.500
7 luteoline glucoside	Lt7gl	24.587
chlorogenic acid	ChA	20.087
caffeic acid	CA	14.587
ferulic acid	FA	28.500

### Antioxidant activity

The Total Phenolic Content, FRAP and DPPH analysis was performed using the Spectrophotometer GENESYS 20 VIS (ThermoScientific, Waltham, USA).

#### Total phenolic content

The following reagents: gallic acid (Sigma-Aldrich, Saint Louis, USA), Folin-Ciocalteu (F-C) reagent (Chempur, Piekary Śląskie, Poland), Sodium carbonate anhydrous, pure per analysis (Chempur, Piekary Śląskie, Poland), methanol, pure per analysis (Polish Reagents, Gliwice, Poland), redistilled water were used in analysis.

#### Ferric ion reducing antioxidant parameter (FRAP)

The ability to reduce iron (III) ions by lavender methanolic extracts was assessed by spectrophotometric method and the following reagents were used:

1. 300 mmol·L^-1^ acetate buffer at pH 3.6, which was prepared by the mixing of 1.6 g sodium acetate trihydrate and 8 mL glacial acetic acid (both reagents are from Polish Reagents, Gliwice, Poland) in 500 mL graduated flask and the distilled water was added to the mark);
2. 10 mmol·L^-1^ TPTZ (tripyrydylo-2,4,6-s-triazine; Merck, Darmstadt, Germany) solution in 40 mmol·L^-1^ hydrochloric acid (Polish Reagents, Gliwice, Poland);
3. 20 mmol·L^-1^ water solution of iron chloride(III) (Chempur, Piekary Śląskie, Poland). From the above reagents the FRAP was prepared in the following way: 416 mL of acetate buffer, 42 mL of TPTZ solution and 42 mL of iron chloride(III) water solution were mixed in 500 mL graduated flask.

Moreover the automatic pipettes 2–20 μL and 100–1000 μL Optipette (HTL, Warsaw, Poland) were also used in experiment.

#### α, α-diphenyl-β-picrylhydrazyl (DPPH) free radical scavenging method

The DPPH (Sigma-Aldrich, Saint Louis, USA) reagent was prepared by solution of 0.0039 g of the substance in methanol (Polish Reagents, Gliwice, Poland) in 100 mL graduated flask and it was stored in dark place.

### Statistical analysis

The obtained HPLC chromatograms of *Lavendulae* methanolic extracts were exported as txt ASCII files and the chemometric process was performed using SpecAlign program (version 2.4.1.) (http://powcs.med.unsw.edu.au/research/adult-cancer-program/services-resources/specalign). The source data are in S1 Table. The 9000 rows and 7 columns matrix was generated and saved as csv (comma-separated values) files. The chromatograms were smoothed using the Savitzky-Golay algorithm and next, the noise was reduced using the discrete waves transformation "Symmlet-8". The baseline was assigned (the window width equal to 20% of length of the chromatogram) and it was removed to elimination the increase resulting from the ‘background’. The alignment of the chromatograms was performed using the recursive alignment by fast Fourier transform algorithm [34,35]. Next, the aligned chromatograms were divided into the segments and synchronized with selected target in all segments [36]. Synchronization was performed with max Shift equal to five. The obtained data were exported as csv to the Microsoft Excel for Windows and the correlation matrix was created.

The Pearson correlation coefficient (r) was used for the evaluation of the chemical similarity between the obtained *Lavandulae* fingerprint chromatograms. It is known, that value of r is from -1 to 1, the high absolute value of r confirms the strong relationship between the data and the zero value show the lack of the correlation.

Moreover the Euclidean distance measure was calculated to confirm the similarity between samples. Euclidean distance is the distance between two points in *n*-dimensional space equal with the length of the segment connecting these points. The value of Euclidean is close to zero for similar vectors.

Principal Component Analysis was performed using the Statistica (version 13.0).

## Results and discussion

### Extraction and HPLC

**Fig 1:**
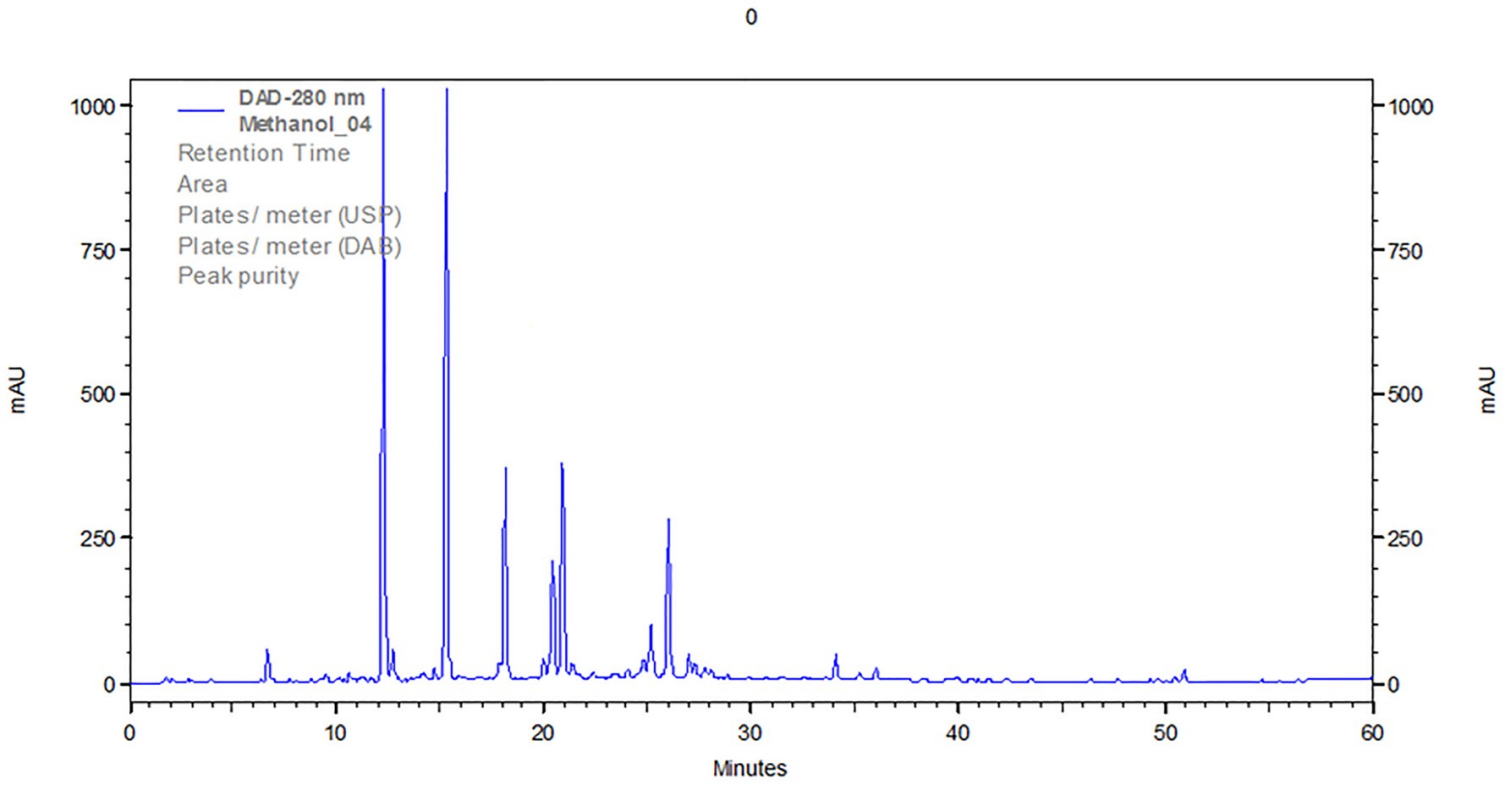
The HPLC chromatogram of methanolic extract of *Lavandula angustifolia „Rosea”*. The abbreviations of standards according to Table 2.

The raw material of selected *Lavandulae* species were weighted (Table 1) and the extraction was performed using xylene and methanol as solvents. The xylene extraction was used to get rid of the ballast substances (e. g. chlorophylls) and only methanolic extracts were further analyzed. The High Performance Liquid Chromatography was performed using the gradient elution with the RP-18 chromatographic column and the mobile phase consisting of methanol, water and 0.1% formic acid by 60 minutes. Selected chromatographic system was used to obtain the fingerprint chromatograms of selected lavenders with the best separation of some phenolic compounds (Table 2), which probably occur in these plants. The exemplary chromatogram of *Lavandula angustifolia „Rosea”* was presented in Fig 1.

The identification of the standards in studied *Lavandulae* species was performed based on the retention time values and the comparison of UV-VIS spectrum of standards and peaks in chromatograms. The presence of selected standards in studied *Lavandulae* species was presented in Table 3. The presence of luteolin 7-glucoside, chlorogenic acid and caffeic acid was confirmed in all studied lavender methanolic extracts.

**Table 3:** The presence of selected standards (Table 2) in studied *Lavandulae* methanolic extracts. Abbreviations of standards according to Table 2.

Name of sample	Standards						
	Ap	Myr	Lt	Lt7gl	ChA	CA	FA
*Lavandula stoechas*	+	-	+	+	+	+	-
*Lavandula lanata*	-	+	+	+	+	+	-
*Lavandula viridis*	-	+	+	+	+	+	+
*Lavandula angustifolia „Rosea”*	+	+	-	+	+	+	-
X	-	-	-	+	+	+	-
*Lavandula angustifolia „Afropurpurea”*	-	-	-	+	+	+	-
*Lavandula angustifolia*	+	-	-	+	+	+	-

Besides, the presence of apigenin was confirmed for *Lavandula stoechas*, *Lavandula angustifolia „Rosea”* and *Lavandula angustifolia*; the presence of myricetin was confirmed for *Lavandula lanata*, *Lavandula viridis* and *Lavandula angustifolia „Rosea”*; luteolin is presented in *Lavandula stoechas*, *Lavandula lanata* and *Lavandula viridis*; ferulic acid is identified for *Lavandula viridis*.

**Fig 2:**
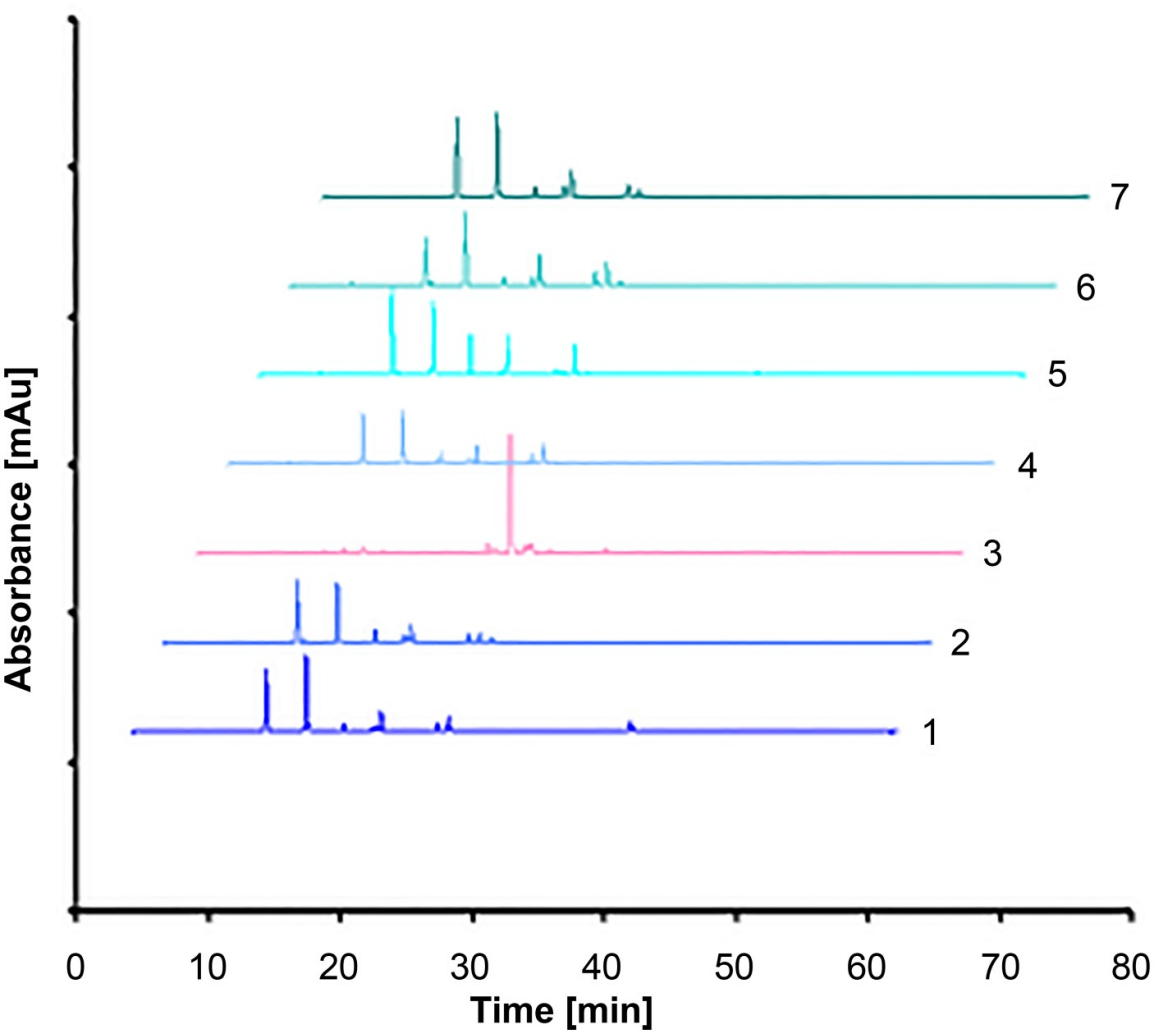
Summarized HPLC chromatograms of selected lavenders (methanolic extracts). Numbers of extracts according to Table 1.

The summarized chromatograms of studied *Lavandulae* methanolic extracts were presented in Fig 2.

### Chemometric analysis

**Fig 3:**
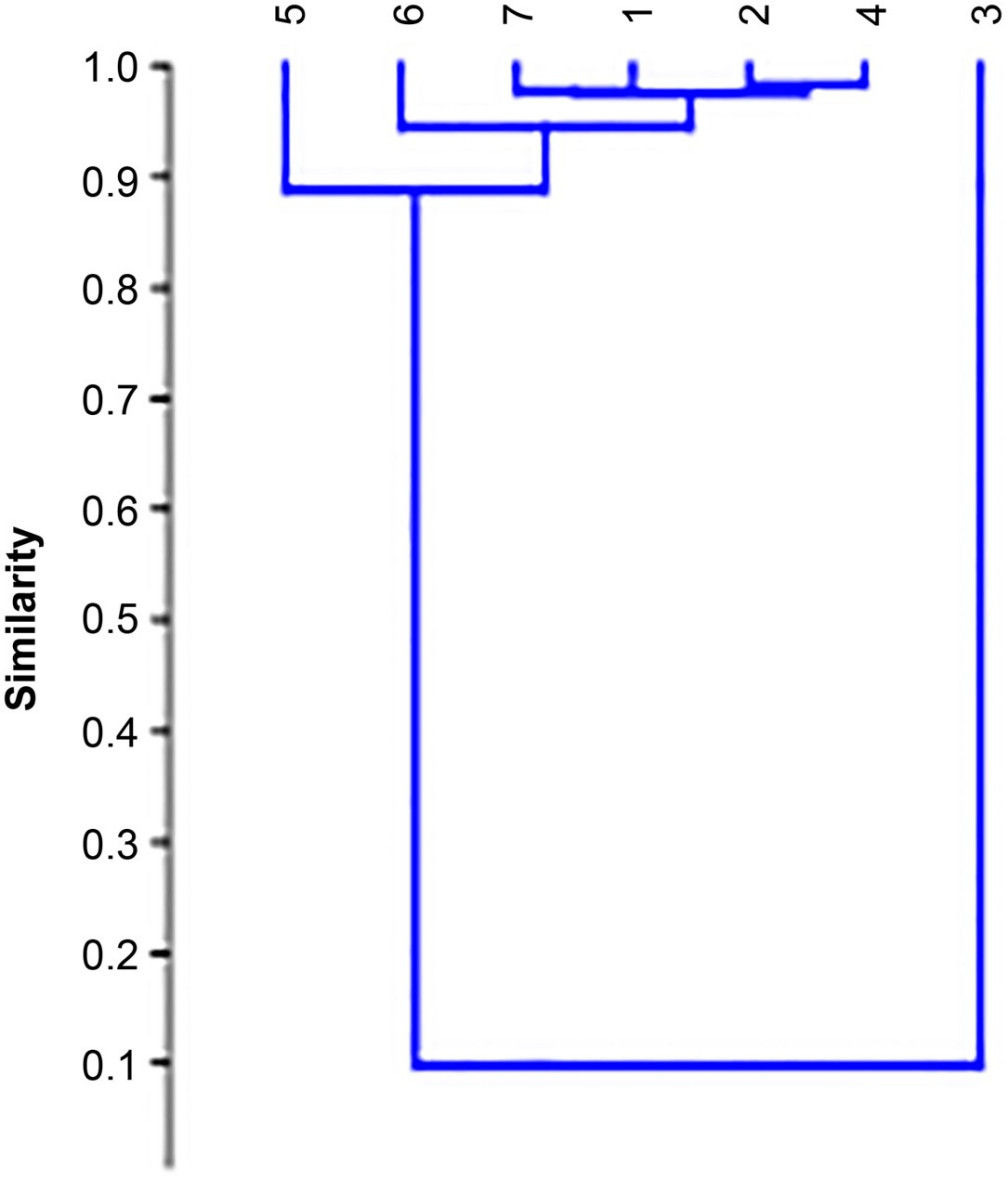
The dendrogram based on Pearson correlation coefficient. Numbers of extracts according to Table 1.

**Fig 4:**
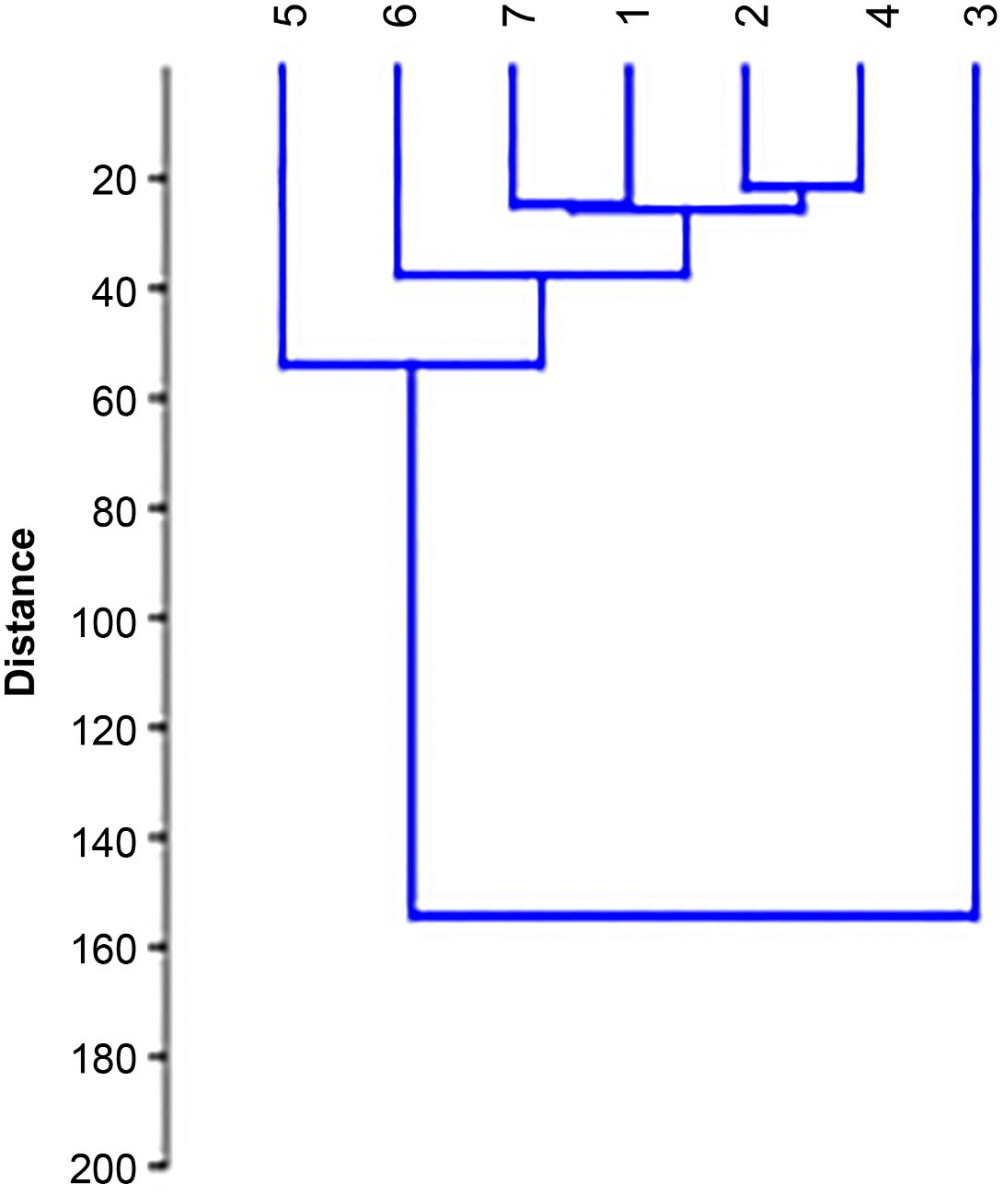
The dendrogram based on Euclidean distance. Numbers of extracts according to Table 1.

The similarity between studied *Lavandulae* species was performed using the chemometric methods (Cluster Analysis and Principal Component Analysis). The similarity dendrograms of selected samples are presented in Figs 3 and 4. The dendrogram based on the Pearson correlation coefficient (similarity index) values is presented in Fig 3 and six samples (except sample 3) are located close to each other (with r equal about 0.9). The highest values of r were obtained for the following pairs: 4 (*Lavandula angustifolia „Rosea”*) and 6 (*Lavandula angustifolia „Afropurpurea”*) with r = 0.9417; for 7 (*Lavandula angustifolia*) and 6 (*Lavandula angustifolia „Afropurpurea”*); and for 2 (*Lavandula lanata*) and 5 (unknown lavender). The sample 3 (*Lavandula viridis*) is most different from the others, the value of r is low (r = 0.2045).

The next dendrogram based on the distance measure (Euclidean distance) is presented in Fig 4. The values of Euclidean are lowest (the highest similarity between samples) for *Lavandula stoechas*, *Lavandula lanata*, *Lavandula angustifolia „Rosea”*, *Lavandula angustifolia „Afropurpurea”*, *Lavandula angustifolia* and one unknown species. The high value of Euclidean was obtained for the sample of *Lavandula viridis* and it confirms its low chemical similarity in comparison with the other samples.

**Fig 5:**
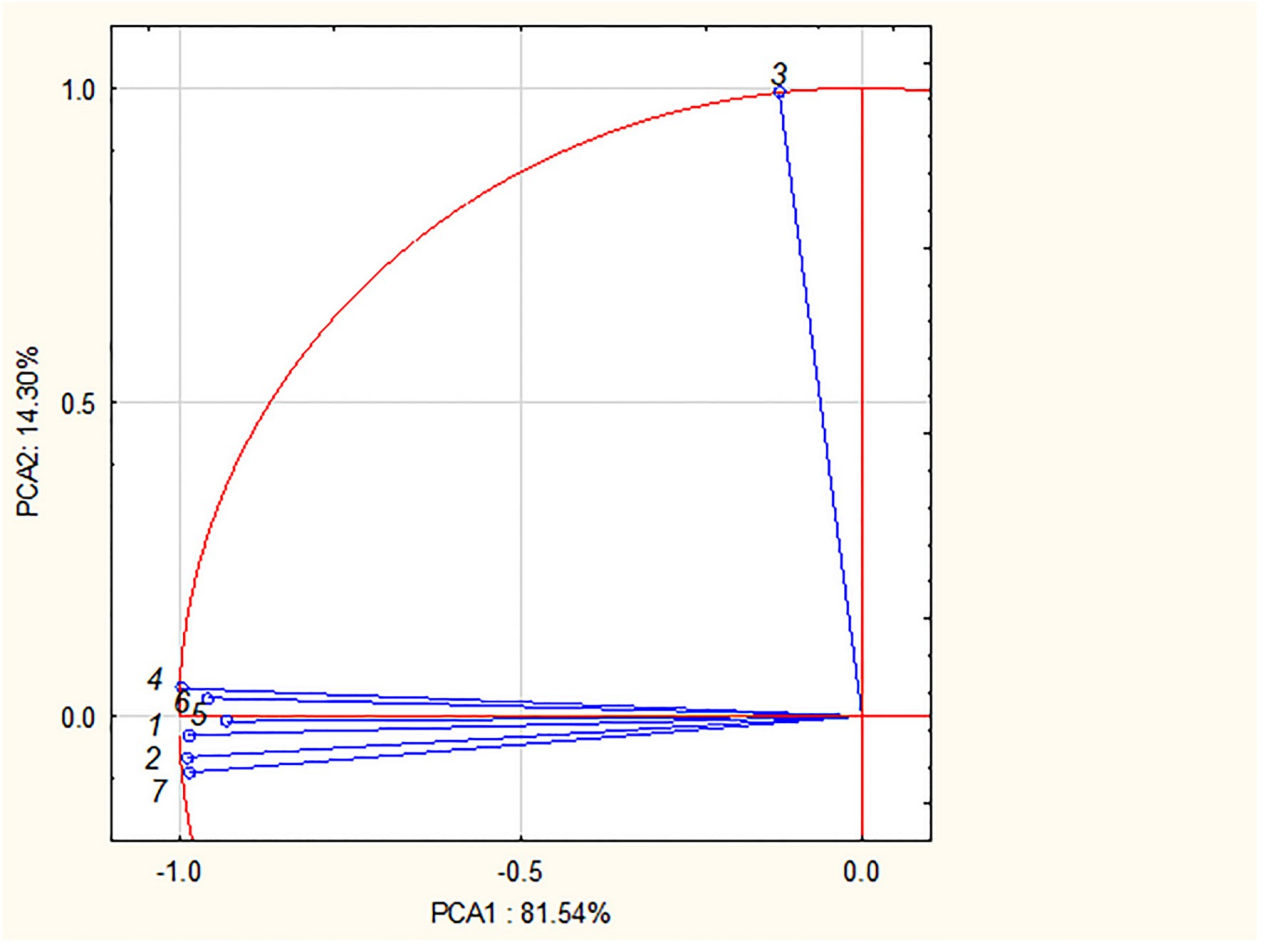
The PC1 vs. PC2 graph for thr chromatographic data of studied samples. Numbers of extracts according to Table 1.

The Principal Component Analysis was also performed and the results were presented as PC1 vs. PC2 graph (Fig 5). The vectors with the appropriate number (according to Table 1) correspond with the studied *Lavandulae* species. The close location of vectors confirms the similarity between selected samples. It is evident, that the following vectors are close to each other: vectors corresponding to samples 1 (*Lavandula stoechas*), 2 (*Lavandula lanata*), 4 (*Lavandula angustifolia „Rosea”*), 6 (*Lavandula angustifolia „Afropurpurea”*), 7 (*Lavandula angustifolia*) and 5 (unknown). The vector corresponding to sample 3 (*Lavandula viridis*) is located far away and it confirms its dissimilarity.

Due to the presence of ferulic acid in the *L*. *viridis* methanolic extract, this sample is chemically different to each other and it was confirmed based on Cluster Analysis and PCA.

### Antioxidant activity

#### Total phenolic content

The Total Phenolic Content analysis was performed using the spectrophotometric method with the Folin-Ciocalteu (F-C) reagent. Gallic acid was used as standard in concentrations (c [mg∙mL^-1^] = 2.0; 1.0; 0.5; 0.25; 0.125; 0.01). 0.01 mL of different concentration of gallic acid solution, 0.99 mL of distilled water and 0.1 mL of F-C reagent were titrated to the tubes and after 5 minutes 1 mL of sodium carbonate (the concentration 75 g∙L^-1^) and 0.4 mL of distilled water were added. Next, the tubes were located in the dark place for two hours. After that, the absorbance was measured at 760 nm for all concentrations of gallic acid. Based on the absorbance measure obtained for gallic acid solutions (A [Au] = 0.691 ±0.001; 0.373 ±0.002; 0.205 ±0.001; 0.105 ±0.001; 0.072 ±0.013, 0.016 ±0.001; respectively) the calibration curve was created with the following equation:

$$y=0.3368∙x+0.0253;R^{2}=0.9984$$

where *y* is absorbance (A), *x* is the concentration of the sample (c).

Next, the absorbance of all obtained *Lavandulae* methanolic extracts was measured under the same conditions as in the case of gallic acid solutions. The values of absorbance of extracts and the values of concentrations (measured and total) read from the calibration curve, are presented in Table 4.

**Table 4:** The absorbance values and the concentration of gallic acid for the total phenolic content (n = 3).

No	Name of sample	Absorbance [Au]^a^	Concentration [mg∙mL^-1^]
1	*L*. *stoechas*	0.575 ±0.000	1.632
2	*L*. *lanata*	0.435 ±0.001	1.216
3	*L*. *viridis*	1.128 ±0.000	3.274
4	*L*. *angustifolia „Rosea”*	0.692 ±0.001	1.980
5	*x*	0.571 ±0.001	1.620
6	*L*. *angustifolia „Atropurpurea”*	0.517 ±0.001	1.460
7	*L*. *angustifolia*	0.490 ±0.001	1.380

^a^standard deviation

All analyzed lavender methanolic extracts show the antioxidant activity measured and calculated using the spectrophotometric F-C method. The total phenolic content was within the range from 1.380–3.274 mg∙mL^-1^. The highest value of absorbance measured (the highest of phenolics content) was obtained for sample 3 (*Lavandula viridis*) and the lowest (the lowest of phenolics content) for sample 2 (*Lavandula lanata*). The absorbance values of the others samples (1, 4, 5, 6, 7 and X) are similar and are between 0.490 and 0.692.

#### Ferric ion reducing antioxidant parameter (FRAP)

The ability to reduce iron(III) ions by lavender methanolic extracts was assessed by spectrophotometric method. The measure of the increase of the absorbance during the reduction of the Fe^3+^ -TPTZ compound (ferric-2,4,6-tripyridyl-s-triazine complex) to Fe^2+^-TPTZ was performed for plant extracts.

At the beginning, the solutions of gallic acid with different concentrations (c [mg∙mL^-1^] = 2.0; 1.0; 0.5; 0.25; 0.125; 0.01) were prepared. 995 μL of FRAP reagent and 5 μL of the different concentrations of gallic acid were measured using an automatic pipette and located in test-tubes. Next, the samples were placed in dark for 8 minutes. After that, the absorbance was measured at 593 nm (Table 5). Based on the absorbance measure obtained for gallic acid solutions (A [Au] = 2.898 ±0.001; 1.819 ±0.002; 1.207 ±0.010; 0.818 ±0.002; 0.577 ±0.010; 0.45 ±0.035; respectively) the calibration curve was created with the following equation:

$$y=1.2772∙x+0.4830;R^{2}=0.9957$$

where *y* is absorbance (A), *x* is the concentration of sample (c).

**Table 5:** The absorbance values and the concentration of gallic acid for the FRAP (n = 3).

No	Name of sample	Absorbance [Au]^a^	Concentration [mg∙mL^-1^]
1	*L*. *stoechas*	2.282 ±0.001	1.409
2	*L*. *lanata*	1.042 ±0.001	0.438
3	*L*. *viridis*	0.852 ±0.001	5.870
4	*L*. *angustifolia „Rosea”*	1.934 ± 0.001	1.136
5	*x*	2.047 ± 0.001	1.225
6	*L*. *angustifolia „Atropurpurea”*	1.684 ±0.001	0.940
7	*L*. *angustifolia*	1.410 ±0.001	0.726

^a^standard deviation

In the same conditions the absorbance values of *Lavandulae* extracts were measured and the results were shown in Table 5. In the case of sample 3 two-fold dilution was made and the absorbance value presented in Table 5 is after the dilution. Based on the obtained results it can be concluded that the highest value of absorbance (and the highest concentration value– 5.870 mg∙mL^-1^) is for the sample 3 (*L*. *viridis*) and the lowest concentration value was obtained for *L*. *lanata* (0.438 mg∙mL^-1^). The concentrations of the other samples are relatively high and their values are between 0.726 and 1.409 mg∙mL^-1^.

#### α, α-diphenyl-β-picrylhydrazyl (DPPH) free radical scavenging method

The absorbance measure was performed with 20 μL of different concentrations (c [mg∙mL^-1^] = 2.0; 1.0; 0.5; 0.25; 0.125; 0.01) of gallic acid solutions and 1.5 μL of DPPH solution. The test-tubes were stored in dark place for 30 minutes. The solution of the radical is dark purple. The ability to scavening free radicals increases with the disappearance of this color, At the beginning the absorbance measure was performed for gallic acid and the calibration curve is as follows:

$$y=-0.3573∙x+0.7960;R^{2}=0.9971$$

where *y* is absorbance (A), *x* is the concentration of sample (c).

The equation was calculated based on the following data: the different concentrations of gallic acid—c [mg∙mL^-1^] = 2.0; 1.0; 0.5; 0.25; 0.125; 0.01 and absorbance values (respectively): 0.095 ±0.003; 0.415 ±0.014; 0.609 ±0.007; 0.705 ±0,005; 0.759 ±0.0006; 0.805 ±0.001.

The values of % inhibition [%] (100· (A0—A)/A_0_) were as follows (respectively): 88.82; 51.18; 28.35; 17.06; 10.71; 5.29. Based on these results the following equation of calibration curve was calculated:

$$y=0.4204∙x+0.0635;R^{2}=0.9971$$

where *y* is % of inhibition, *x* is the absorbance (A).

Next, the absorbance measure for the analyzed *Lavandulae* extracts was performer in the same conditions as gallic acid. The absorbance measure values, the concentration and % inhibition of studied extracts were presented in Table 6. Sample 3 (*L*. *viridis*) was two-fold diluted and the results presented in Table 6 are after dilution. Based on the results it can be concluded that all samples show the antioxidant activity, the highest inhibition % was obtained for *L*. *viridis* (99.47%) and the lowest for *L*. *angustifolia* (91.51%). The % inhibition of the other samples are between 92.09–95.18%.

**Table 6:** The absorbance values and % of inhibition for the DPPH (n = 3).

No	Name of sample	Absorbance [Au]^a^	inhibition% [%]
1	*L*. *stoechas*	0.042 ±0.000	95.18
2	*L*. *lanata*	0.063 ±0,003	92.78
3	*L*. *viridis*	0.053 ±0,001	99.47
4	*L*. *angustifolia „Rosea”*	0.053 ±0.000	93.92
5	*x*	0.043 ±0.001	95.07
6	*L*. *angustifolia „Atropurpurea”*	0.069 ±0.010	92.09
7	*L*. *angustifolia*	0.074 ±0,006	91.51

^a^standard deviation

In conclusion, all *Lavandulae* extracts show the antioxidant activity and the highest ability to scavenging free radicals was obtained for *L*. *viridis* and the lowest for *L*. *angustifoliae*.

## Conclusions

The methanolic extracts of seven *Lavandulae* species (*L*. *stoechas*, *L*. *lanata*, *L*. *viridis*, *L*. *angustifolia „Rosea”*, *L*. *angustifolia „Afropurpurea”*, *L*. *angustifolia* and one unknown) were analyzed using RP-HPLC–DAD gradient elution technique to obtain the chromatographic fingerprint profiles. The chromatographic data were processed using the SpecAlign program.

The presence of luteolin 7-glucoside, chlorogenic acid and caffeic acid was confirmed in all studied lavender methanolic extracts. Moreover, the presence of apigenin was confirmed for *L*. *stoechas*, *L*. *angustifolia „Rosea”* and *L*. *angustifolia*; the presence of myricetin was confirmed for *L*. *lanata*, *L*. *viridis* and *L*. *angustifolia „Rosea”*; luteolin is presented in *L*. *stoechas*, *L*. *lanata* and *L*. *viridis*; ferulic acid is identified for *L*. *viridis*.

The obtained chromatograms were compared using the Cluster Analysis and PCA and in conclusion the most of studied samples (except to *L*. *viridis*) were chemically similar. The unknown *Lavandulae* species was chemically similar to the most of studied samples (except to *L*. *viridis*).

*L*. *viridis* methanolic extract is chemically different to each other due to the presence of ferulic acid.

The preliminary evaluation of the antioxidant activity of selected *Lavandulae* species based on the total phenolic content, FRAP and DPPH was performed. In all examined methanolic extracts the presence of phenolics and the ability to scavenging free radicals was observed. The total phenolic content was within the range from 0.10–1.36 mg∙cm^-3^ and the highest value of absorbance was obtained for sample 3 (*Lavandula viridis*) and the lowest for sample 7 (*Lavandula angustifolia*) and 2 (*Lavandula lanata*).

Based on FRAP results, the highest value of absorbance (and the highest concentration– 5.870 mg∙cm^-3^) is for the sample 3 (*L*. *viridis*) and the lowest concentration was obtained for *L*. *lanata* (0.438 mg∙cm^-3^). Similarly results were obtained for DPPH method, where the highest inhibition % was obtained for *L*. *viridis* (99.47%) and the lowest for *L*. *angustifolia* (91.51%).

## Supporting information

S1 Table Source data for chemometric calculations. Wavelength 280 nm.
(XLSX)
